# Cognitive load and teachers’ innovative behavior in AI-enhanced English language instruction: A mediation analysis of technological adaptability

**DOI:** 10.1371/journal.pone.0343002

**Published:** 2026-03-05

**Authors:** Su Dongying, Meijuan Gai, Aikebaier Simayilijiang

**Affiliations:** 1 School of Foreign Language, Ningxia Medical University, Yinchuan City, Ningxia Province, China; 2 Shandong Yingcai University, Jinan City, Shandong Province, China; 3 Academy of Language Studies, Universiti Teknologi MARA, Selangor, Malaysia; 4 School of Foreign Studies, North Minzu University, Yinchuan City, Ningxia Province, China; University of Tartu, ESTONIA

## Abstract

This investigation examines the complex interrelationships between teachers’ cognitive load, technological adaptability, and innovative teaching behavior in AI-enhanced educational environments. Through the integration of Cognitive Load Theory, Technology Acceptance Model, and Innovation Diffusion Theory, we develop a sophisticated theoretical framework for understanding how cognitive demands influence teaching innovation through adaptive mechanisms. Employing exploratory structural equation modeling with WLSMV estimation (N = 600), we analyze data collected through rigorously validated instruments measuring AI-assisted Teaching Cognitive Load (ATCL), technological adaptability, and innovative teaching behavior. Results reveal a significant negative relationship between cognitive load and innovative teaching behavior (*β* = −.134, p < .001), mediated by technological adaptability (indirect effect *β* = −.171, p < .001). The measurement model demonstrates exceptional psychometric properties (α = .91−.93; AVE = .64−.68) and establishes measurement invariance across teacher subgroups (ΔCFI ≤ .001). These findings advance theoretical understanding of cognitive-adaptive mechanisms in technology-enhanced teaching while providing empirically validated pathways for enhancing pedagogical innovation. The study contributes methodologically through the development of the ATCL scale and analytically through sophisticated mediation analysis techniques. Implications extend to professional development strategies, institutional policy formulation, and the theoretical conceptualization of cognitive load in AI-enhanced educational environments.

## 1. Introduction

The integration of artificial intelligence (AI) into educational ecosystems represents a transformative shift in teaching practices, fundamentally altering pedagogical approaches in language instruction. This transformation, occurring at the intersection of cognitive psychology, educational technology, and pedagogical innovation, requires understanding the interplay between teachers’ cognitive processes, adaptive capabilities, and innovative practices. The theoretical framework underpinning this investigation synthesizes Cognitive Load Theory (CLT), Technology Acceptance Model (TAM), and Innovation Diffusion Theory (IDT) to examine the dynamics of AI integration in educational contexts.

The theoretical foundation for examining teachers’ cognitive processes during AI integration stems from Cognitive Load Theory. While CLT’s initial conceptualization [1] focused on learner cognition, its scope has expanded to encompass instructional design and delivery [2]. Contemporary interpretations of CLT, particularly in technology-enhanced environments, posit that educators’ cognitive resources are allocated across multiple dimensions: the intrinsic complexity of AI systems, the extraneous load of interface navigation, and the germane load associated with pedagogical integration [3].

These cognitive considerations intersect with technological adaptability through Teacher Cognitive Adaptability Theory, which links educators’ instructional flexibility to their cognitive processing capabilities. This perspective is complemented by the Technology Acceptance Model, which explains psychological mechanisms underlying technology adoption, and Innovation Diffusion Theory, which addresses social and systemic factors influencing technological integration. Recent empirical investigations have documented the relationships between cognitive demands and pedagogical innovation in AI-enhanced educational environments [4]. This transformation is particularly relevant in English language instruction, where AI integration demands attention to linguistic pedagogy, technological proficiency, and innovative teaching methodologies.

Despite extensive documentation of AI implementation in educational settings, several critical research gaps persist. First, the cognitive mechanisms underlying successful technology adoption remain inadequately theorized, particularly regarding the relationships between cognitive load, technological adaptability, and innovative teaching practices. Second, the pathways through which cognitive load influences technological adaptability and innovative behavior have not been systematically investigated. While existing research acknowledges the importance of adaptive capabilities [5], the mediating processes remain obscured by methodological limitations and theoretical fragmentation. Third, current analytical approaches have proven insufficient for examining the multidimensional relationships between cognitive processes and pedagogical innovation, impeding the development of comprehensive theoretical models to inform professional development strategies.

This study examines the relationships between teachers’ cognitive load, technological adaptability, and innovative teaching behavior in AI-assisted English education through mediation analysis. The investigation addresses three key research questions: (1) How do variations in cognitive load influence teachers’ innovative behavior in AI-assisted English teaching contexts, considering both direct and indirect pathways? (2) To what extent does technological adaptability mediate the relationship between cognitive load and innovative teaching behavior, and what are the underlying psychological mechanisms facilitating this mediation? (3) What specific cognitive and adaptive processes facilitate or inhibit innovative teaching practices in AI-enhanced educational environments, and how can these insights inform professional development interventions?

This research contributes to both theoretical advancement and practical application in AI-assisted education. Theoretically, it integrates cognitive load theory with technological adaptability models, offering a nuanced understanding of innovation in educational contexts. Methodologically, it demonstrates the utility of mediation analysis in examining complex educational phenomena. Practically, the findings inform the development of evidence-based interventions for enhancing teachers’ technological adaptability and innovative capabilities in AI-enhanced educational environments.

## 2. Literature review and theoretical framework

### 2.1. Cognitive load theory in AI-enhanced education

#### 2.1.1. Theoretical evolution and contemporary extensions.

Cognitive Load Theory (CLT) underwent significant theoretical evolution since its initial conceptualization by Sweller [1], transitioning from a narrow focus on problem-solving cognition to a comprehensive framework for understanding complex instructional environments. The theory’s contemporary interpretation, as articulated by Sweller, Ayres, and Kalyuga [6], provided sophisticated analytical tools for examining the multifaceted cognitive demands imposed by technological integration in pedagogical contexts. This theoretical progression reflected a crucial paradigm shift from viewing cognitive load as a unitary construct to understanding it as a complex, interactive phenomenon particularly relevant in AI-enhanced educational environments.

Chandler and Sweller’s [3] foundational work established the tripartite framework of intrinsic, extraneous, and germane cognitive load, which has proved instrumental in analyzing teachers’ cognitive processes during technology integration. Recent theoretical advances, particularly Skulmowski and Xu’s [7] reconceptualization of extraneous cognitive load in digital environments, suggested that traditional CLT frameworks require substantial adaptation to fully capture the unique cognitive demands of AI-enhanced teaching contexts. This theoretical refinement emphasizes the dynamic interplay between different types of cognitive load, challenging earlier linear conceptualizations.

#### 2.1.2. Empirical validation and methodological advances.

Empirical investigations significantly enhanced our understanding of cognitive load in educational technology contexts. Dalinger’s [8] development of instruments measuring teachers’ cognitive load during technology adoption represented a crucial methodological advancement, demonstrating that cognitive load manifested through complex interactions rather than simple additive effects. This finding had profound implications for understanding how teachers process and integrate AI-based instructional tools.

### 2.2. Technology acceptance and teacher adaptability

#### 2.2.1. Theoretical foundations and extensions.

The Technology Acceptance Model (TAM) provided foundational framework for understanding teachers’ adaptive responses to AI integration. Davis’s [9] seminal work established perceived usefulness and ease of use as core determinants of technology adoption, while Venkatesh and Davis’s [10] extensions incorporated social influence and cognitive instrumental processes specific to professional contexts. Recent applications to generative AI contexts revealed that traditional TAM constructs required substantial modification. Gong, Xu, Luo, and Lin’s [11] structural equation modeling analysis demonstrated that LLM adoption among teacher education students depends critically on perceived anthropomorphism and conversational quality beyond conventional usefulness constructs, suggesting that human-like AI interactions introduced novel psychological dynamics absent in earlier educational technologies.

#### 2.2.2. Contemporary applications in AI-enhanced education.

Empirical investigations of generative AI acceptance reveal complex patterns across institutional contexts. [12] investigation of EFL teachers established use experience as critical moderator, with hands-on engagement significantly strengthening perceived usefulness-adoption intention relationships. Chen's [13] analytic hierarchy process study identified technological competence and institutional support as primary adoption determinants, demonstrating that acceptance transcends individual psychological factors to encompass organizational infrastructure and professional development resources.

Critical for the present investigation, Zhang et al.’s [14] quasi-experimental evaluation of AI-driven feedback tools established empirical linkages between cognitive load and technology acceptance. Instructors reporting higher extraneous cognitive load during initial implementation demonstrated significantly lower acceptance levels, with perceived pedagogical effectiveness mediating this relationship. This finding provided empirical precedent for examining cognitive load as antecedent to technology acceptance and adaptive behavior in AI-enhanced educational environments. These contemporary investigations collectively demonstrate that while TAM’s foundational constructs maintained explanatory power, generative AI technologies’ distinctive characteristics necessitate theoretical extensions incorporating AI-specific factors including anthropomorphism, output reliability, institutional support, and cognitive processing demands examined in this study.

### 2.3. Innovation diffusion and teaching behavior

#### 2.3.1. Historical development and theoretical evolution of IDT.

Innovation Diffusion Theory (IDT) has evolved substantially since Rogers’ [15] seminal work, progressing from a descriptive model to a robust explanatory framework. Rogers’ initial conceptualization identified five adopter categories (innovators, early adopters, early majority, late majority, and laggards) and traced innovation adoption through awareness, interest, evaluation, trial, and adoption stages. Over subsequent decades, IDT has been empirically validated across diverse domains, with educational applications emerging as a particularly productive area of inquiry. Rogers’ [16] later refinements incorporated key attributes influencing adoption rates—relative advantage, compatibility, complexity, trialability, and observability—that directly inform our understanding of cognitive barriers to technology integration.

The complexity attribute merits particular attention in the context of our research, as it directly corresponds to cognitive load constructs. Early educational applications of IDT by Hall and Hord [17] established the Concerns-Based Adoption Model, which documented how teachers’ concerns about cognitive demands significantly influenced technology adoption patterns. This theoretical development paralleled advances in cognitive load research, creating natural conceptual linkages between the two frameworks that inform our integrated approach. Fullan’s [18] work further established the relationship between perceived complexity and implementation success, demonstrating how cognitive demands influence adoption outcomes in educational settings.

The evolution of IDT has progressively moved toward greater integration with cognitive frameworks. Moore and Benbasat’s [19] extensions incorporated the perceived ease of use construct—directly paralleling elements of the Technology Acceptance Model—while highlighting how cognitive perceptions influence diffusion patterns. Venkatesh et al.’s [20] Unified Theory of Acceptance and Use of Technology further synthesized IDT with elements from cognitive models, creating theoretical precedent for our integrated approach. This historical trajectory demonstrates IDT’s progressive convergence with cognitive frameworks, justifying its inclusion in our theoretical model.

#### 2.3.2. Empirical evidence linking IDT to cognitive load and teaching innovation.

The empirical literature demonstrates clear connections between IDT constructs and cognitive load in educational technology contexts. Sahin’s [21] systematic review of 45 IDT applications in educational settings established complexity perception (a cognitive variable) as a primary determinant of adoption success, explaining 49–87% of variance across multiple studies. This review documented how cognitive demands systematically influence technology diffusion patterns, providing empirical justification for examining cognitive load as a predictor of innovative teaching behavior. Subsequent meta-analytic work by Straub [22] further established how cognitive processing requirements influence technology adoption trajectories across diverse educational settings.

The relevance of IDT to our research questions is further reinforced by empirical investigations demonstrating bidirectional relationships between diffusion patterns and cognitive load. Surry and Ely’s [23] longitudinal studies of educational technology adoption documented how initial cognitive demands gradually diminish through collective adaptation processes, suggesting that diffusion mechanisms may moderate cognitive load effects. This finding directly informs our hypothesis regarding the mediating role of technological adaptability, as it suggests adaptation processes that potentially mitigate cognitive barriers to innovation.

Recent empirical work specifically examining AI integration in educational contexts further validates IDT’s relevance to our research questions. Wu et al.’s [24] investigation of factors influencing rural teachers’ innovative behavior identified cognitive complexity as a critical determinant of AI adoption, establishing direct empirical connections between our cognitive load constructs and innovation outcomes. Similarly, Li and Zhu’s [25] study demonstrated significant relationships between cognitive demands, adaptive responses, and innovative teaching behaviors, providing empirical precedent for our hypothesized mediation model.

#### 2.3.3. Theoretical integration: Bridging cognitive load, adaptability, and innovation.

The integration of IDT with cognitive load theory and technology acceptance models provided essential theoretical mechanisms for understanding how cognitive demands influence teaching innovation through adaptive processes. While cognitive load theory explains the internal cognitive constraints that teachers experience when engaging with AI technologies, IDT illuminates how these constraints manifest in observable adoption patterns and innovative behaviors. This integration addresses a critical theoretical gap in understanding the transition from cognitive experience to behavioral outcomes.

The complexity attribute in IDT directly parallels cognitive load constructs, providing a theoretical bridge between internal processing demands and observable innovation patterns. Rogers’ [26] specification that complexity perception negatively influences adoption rates corresponds to our hypothesis that cognitive load negatively influences innovative teaching behavior. Similarly, IDT’s emphasis on adaptation processes during implementation aligns with our technological adaptability construct, providing theoretical justification for its hypothesized mediating role.

This theoretical integration is particularly relevant in AI-enhanced educational environments, where cognitive demands are often substantial due to the novelty and complexity of AI systems. The IDT framework provided essential explanatory mechanisms for understanding how cognitive load influences innovation patterns, while also identifying potential intervention points for enhancing technological adaptability. This theoretical comprehensiveness is critical for addressing our research questions regarding the relationships between cognitive load, technological adaptability, and innovative teaching behavior in AI-enhanced educational contexts.

### 2.4. Methodological advances in educational technology research

#### 2.4.1. Analytical frameworks and measurement development.

Recent methodological advances, particularly in structural equation modeling [27] and multiple mediation analysis, have enabled more nuanced understanding of the complex relationships between cognitive load, adaptability, and innovation. Teo and Tsai’s [28] methodological framework provided sophisticated analytical tools for examining these intricate pathways, while In’nami and Koizumi’s [29] work established rigorous standards for educational research methodology.

#### 2.4.2. Measurement validation and analytical sophistication.

The development and validation of measurement instruments has become increasingly sophisticated, as evidenced by the emergence of multi-dimensional constructs and advanced psychometric techniques. This methodological evolution facilitates more precise investigation of the complex relationships between cognitive, behavioral, and institutional variables in AI-enhanced educational settings.

### 2.5. Theoretical integration and research gaps

#### 2.5.1. Theoretical mechanisms: Bridging internal cognition and external adaptation.

**Fig 1:**
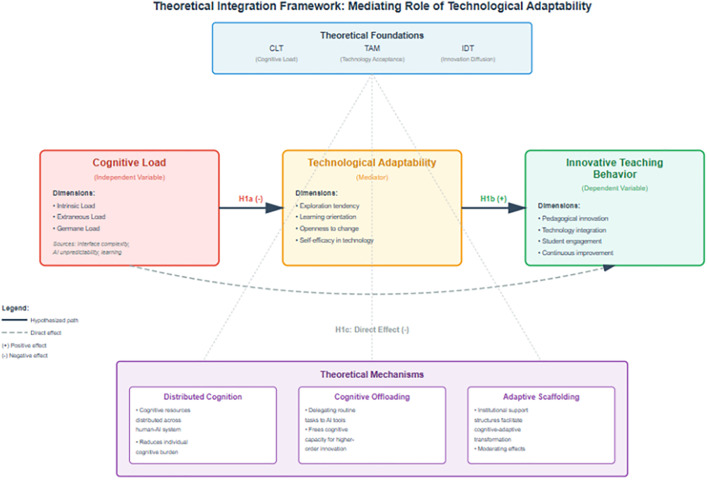
Theoretical integration framework. Theoretical Integration Framework: Mediating Role of Technological Adaptability in the Relationship between Cognitive Load and Innovative Teaching Behavior in AI-Enhanced Educational Contexts. This conceptual model illustrates the integration of Cognitive Load Theory (CLT), Technology Acceptance Model (TAM), and Innovation Diffusion Theory (IDT) through three theoretical mechanisms: distributed cognition, cognitive offloading, and adaptive scaffolding. The model depicts technological adaptability as a mediator between cognitive load and innovative teaching behavior, with specific hypotheses (H1a, H1b, H1c) representing the direct and indirect pathways examined in this study.

The integration of Cognitive Load Theory, Technology Acceptance Model, and Innovation Diffusion Theory reveals a fundamental theoretical challenge: elucidating how cognitive load—an internally-determined phenomenon rooted in working memory constraints—can be mediated by external technological factors. This apparent paradox necessitates examining contemporary theoretical frameworks that bridge cognitive and socio-technical domains (Fig 1).

Distributed cognition theory provided the primary theoretical mechanism for understanding this mediation process ([30]). Within AI-enhanced educational environments, cognitive processes extend beyond individual mental architectures to encompass technological systems that function as integrated components of distributed cognitive networks. Technological adaptability, in this framework, represents educators’ capacity to effectively orchestrate these human-AI cognitive assemblages, transforming isolated cognitive constraints into distributed processing systems [31]. This theoretical perspective fundamentally reconceptualizes cognitive load from a purely internal limitation to a dynamically negotiated phenomenon spanning human and technological agents.

The cognitive offloading framework offers complementary explanatory mechanisms (Gerlich [32]). When educators develop technological adaptability, they acquire capabilities to strategically externalize cognitive functions onto AI systems, thereby restructuring internal processing demands. This process operates through metacognitive monitoring and strategic resource allocation, as demonstrated in recent investigations of AI-mediated instruction (Zhai & Nezakatgoo [33]. Technological pedagogical reasoning competency emerged as a critical mediating construct, suggesting that adaptability encompassed both technical proficiency and metacognitive awareness of cognitive distribution opportunities.

Recent theoretical syntheses reveal bidirectional relationships between cognitive experiences and adaptive responses Gerlich [34]. Initial encounters with AI-induced cognitive demands trigger compensatory adaptation mechanisms, which subsequently reshape cognitive processing patterns through iterative human-AI interaction cycles. This dynamic interplay, documented across diverse educational contexts Glickman & Sharot [35], suggested that technological adaptability not only responded to cognitive load but actively reconfigured it, creating recursive feedback loops that progressively transform teaching practices.

The institutional dimension introduces additional theoretical complexity. Tsakeni [36] conceptualize “adaptive scaffolding” as organizational structures that facilitate cognitive-technological integration, while Zhang [37] identify contextual moderators influencing mediation strength. These findings align with socio-technical systems theory, suggesting that cognitive-adaptive processes are embedded within broader institutional ecologies that either facilitate or constrain adaptation pathways Carayon et al. [38].

#### 2.5.2. Research gaps and theoretical hypotheses.

Despite theoretical advances, critical gaps persist in understanding cognitive-adaptive mechanisms within AI-enhanced educational contexts. First, while associations between cognitive load and technological adaptability have been established (Zhu et al. [39]), the precise causal mechanisms and temporal dynamics remain inadequately theorized. Existing frameworks insufficiently address how different cognitive load types—intrinsic, extraneous, and germane—differentially interact with adaptive processes, particularly in rapidly evolving AI environments [40].

Second, the mediating role of technological adaptability requires more nuanced theoretical specification. Current conceptualizations inadequately distinguish between reactive adaptation (responding to cognitive demands) and proactive adaptation (anticipating and preventing cognitive overload). Furthermore, the cognitive offloading literature has not been systematically integrated with educational technology frameworks, leaving theoretical gaps in understanding how teachers strategically distribute cognitive resources across human-AI assemblages ，For example, distributed cognition and cognitive offloading research highlights how modern technologies can offload cognitive tasks and influence mental processing [41,42].

Third, contextual and individual moderators remain undertheorized. While institutional support structures and professional autonomy are recognized as influential factors, their specific mechanisms of action within the cognitive-adaptive framework require clarification. Similarly, individual differences in working memory capacity, technological self-efficacy, and metacognitive capabilities likely moderate mediation pathways, yet these interactions remain empirically unexplored within AI-enhanced teaching contexts.

Fourth, the implementation stage dimension introduces temporal complexity that current static models inadequately capture. The relationship between cognitive load and innovation likely evolves as teachers progress through adoption stages, yet existing research predominantly employs cross-sectional designs that cannot capture these developmental trajectories. Understanding how cognitive-adaptive processes unfold across implementation phases is essential for developing targeted interventions.

These theoretical considerations and empirical gaps inform the following hypotheses:

H1: Teachers’ cognitive load has a direct negative effect on innovative teaching behavior in AI-enhanced environments, mediated by their technological adaptability.
H2: The relationship between cognitive load and technological adaptability is moderated by institutional support structures and professional autonomy.
H3: The indirect effect of cognitive load on innovative teaching behavior through technological adaptability varies systematically across different stages of AI implementation and adoption.

## 3. Methodology

### 3.1. Research design and sample characteristics

This investigation employed a cross-sectional research design within the framework of a broader research initiative examining technology integration in Chinese higher education (Project ID: NSSFC-21BYY092). The study was conducted between September and December 2023 across 15 higher education institutions in eastern and northwestern China, selected to represent diverse institutional contexts and varying levels of AI technology implementation. Employing a multistage stratified random sampling approach, institutions were stratified by type, region, and level of AI technology implementation, followed by systematic sampling of English language departments and individual teachers. Sample size determination employed Monte Carlo simulation techniques based on pilot studies (n = 45), ensuring sufficient statistical power (1-*β* > .90) for detecting hypothesized relationships.

The analytical sample comprised 600 English language teachers (female: 62%; male: 38%; mean age: 38.6 years, *SD* = 8.3) with teaching experience ranging from 1 to 35 years (M = 12.4, *SD* = 7.8), distributed across experience levels (1–5 years: 27%; 6–10 years: 33%; 11–15 years: 22%; > 15 years: 18%). Participants represented diverse educational contexts (public universities: 68%; private institutions: 32%) and varied levels of prior AI technology experience (beginner: 32%; intermediate: 45%; advanced: 23%). The institutional contexts reflected systematic variation in technological infrastructure, with 28% reporting comprehensive AI integration platforms, 46% reporting partial implementation, and 26% reporting pilot-stage implementation.

### 3.2. Instrumentation and measurement development

The measurement instruments employed in this study were systematically adapted from established scales with demonstrated psychometric properties, followed by a rigorous validation process to ensure contextual appropriateness for AI-enhanced English language teaching environments. Adaptation procedures for all instruments followed a standardized protocol comprising: (1) initial item selection and modification based on theoretical considerations, (2) cognitive interviewing with experienced educators, (3) expert panel evaluation, and (4) pilot testing with subsequent psychometric analysis.

The AI-assisted Teaching Cognitive Load (ATCL) scale was adapted from Dalinger’s [8] Teacher Technology Cognitive Load Instrument and Leppink et al.’s Cognitive Load Instrument. Cognitive interviews conducted with 12 experienced educators employed think-aloud protocols during scale completion (60–90 minutes per interview), followed by retrospective probing regarding item interpretation. Expert review involved five educational technology specialists and three language teaching experts who evaluated item relevance and clarity using standardized rating forms (inter-rater agreement threshold: 85%). The final ATCL scale comprised three subscales capturing distinct dimensions of cognitive load: Intrinsic Load (6 items, α = .88), Extraneous Load (7 items, α = .90), and Germane Load (5 items, α = .86).

The Technological Adaptability measure integrated items from Ployhart and Bliese’s [43] Adaptive Performance Scale, Parasuraman & Colby 's [44] Technology Readiness Index, and Mishra and Koehler’s [45] TPACK instrument. Following similar validation procedures (cognitive interviews with 8 educators, expert review by 6 specialists), the final measure comprised four subscales: Cognitive Flexibility (4 items, α = .85), Innovation Orientation (4 items, α = .87), Uncertainty Management (4 items, α = .84), and Integration Capability (4 items, α = .89).

The Innovative Teaching Behavior scale was adapted from Thurlings et al.'s [46] Teacher Innovative Behavior Instrument and Janssen’s [47] Innovative Work Behavior measure. Adaptation involved cognitive interviews with 10 English teachers and expert review by a panel of 7 specialists. The final scale comprised three subscales: Idea Generation (5 items, α = .86), Idea Promotion (5 items, α = .88), and Idea Implementation (5 items, α = .90).

All scales employed 7-point Likert response formats, with construct validity established through confirmatory factor analysis. The measurement validation process demonstrated robust psychometric properties across all instruments, with reliability coefficients exceeding conventional thresholds and factor structures aligning with theoretical expectations.

### 3.3. Data collection and analytical strategy

Data collection proceeded according to a standardized protocol to ensure measurement consistency across institutional contexts. Participants were recruited through institutional coordinators and accessed the survey through a secure online platform between September and December 2023. Methodological features to enhance data quality included strategically positioned attention check items (n = 4), page timing metrics to identify excessively rapid completion, and randomized item presentation within scales to mitigate order effects. The survey required approximately 25–35 minutes to complete based on pilot testing and actual completion metrics.

The analytical framework employed structural equation modeling with weighted least squares mean and variance adjusted (WLSMV) estimation, selected for its superior performance with ordinal data and non-normal distributions. Analysis proceeded through a two-stage approach, examining the measurement model before testing structural relationships. Preliminary data screening revealed minimal missing data (<2% per variable) determined to be MCAR (χ^2^ = 135.42, df = 128, p = .312), permitting the use of full information maximum likelihood estimation.

Measurement model evaluation employed confirmatory factor analysis with multiple fit indices (CFI, TLI, RMSEA, SRMR), using established thresholds for acceptable fit [48]. Reliability was assessed through multiple indicators, including Cronbach’s alpha, composite reliability, and average variance extracted, while discriminant validity was evaluated by comparing the square root of AVE values with inter-construct correlations.

The structural model examined hypothesized relationships among latent constructs, with particular focus on the mediating role of technological adaptability in the relationship between cognitive load and innovative teaching behavior. Mediation analysis employed the product of coefficients approach with bias-corrected bootstrap confidence intervals (5,000 resamples) to test the significance of indirect effects. While our conceptual model specified technological adaptability as a mediator, the multidimensional nature of both cognitive load and technological adaptability created a complex mediation structure. This analysis is characterized as a mediation analysis with multidimensional constructs rather than a multiple mediation analysis with parallel mediators, examining specific indirect effects through each dimension while controlling for other dimensions.

Measurement invariance testing examined whether the measurement model functioned equivalently across teacher subgroups defined by teaching experience and institutional type, proceeding through a sequential approach testing increasingly restrictive models (configural, metric, scalar, and structural invariance). Changes in fit indices (ΔCFI ≤ .01, ΔRMSEA ≤ .015) across increasingly constrained models were used to evaluate invariance, following Chen’s [13] recommendations. All analyses were conducted using Mplus version 8.3, with supplementary analyses performed in R version 4.0.3.

To assess potential common method bias arising from single-source self-report data, we conducted Harman’s single-factor test by entering all measurement items into an exploratory factor analysis without rotation. The results indicated that the first unrotated factor accounted for 40.2% of variance, below the 50% threshold, suggesting that common method bias is not a major concern in this study [49]. This finding, combined with procedural remedies implemented during data collection (e.g., guaranteed anonymity, counterbalanced item order), provides reasonable confidence that common method variance does not substantially threaten the validity of our findings.

### 3.4. Ethical approval and research conduct

Ethical approval for this study was granted by the Institutional Review Board of Ningxia Medical University (approval No. NXMU-IRB-20230821) on August 21, 2023, with validity through August 20, 2024. The IRB review confirmed that the study protocol met all ethical standards stipulated by relevant national and institutional guidelines, including protection of human subjects, informed consent procedures, confidentiality protocols, and risk minimization measures.

All research procedures adhered to the Declaration of Helsinki and PLOS ONE guidelines for research involving human participants. In-service English teachers aged 18 years or older were prospectively recruited through institutional coordinators between September 1 and December 31, 2023. Participation was entirely voluntary, with no minors involved in the study. Before accessing the anonymous, secure online questionnaire, each participant reviewed an electronic information sheet detailing the study’s purpose, procedures, potential risks and benefits, and data handling protocols. Written informed consent was obtained through an electronic “I agree” button, confirming participants’ voluntary agreement to participate.

The study involved no invasive procedures, biomedical interventions, or high-risk protocols. All data were collected anonymously, with no personally identifiable information recorded at any stage of the research process. This ensured that investigators had no access to individually-identifiable data throughout data collection, analysis, and reporting. All collected data are stored securely and used solely for academic and research purposes in accordance with institutional data protection policies.

## 4. Results

### 4.1. Measurement validation

Preliminary analyses established robust psychometric properties across all theoretical constructs. To establish the basic characteristics of our measures, we first examined descriptive statistics. Table 1 reveals satisfactory descriptive statistics, with means ranging from 3.84 (ATCL) to 4.12 (TA), and standard deviations demonstrating appropriate score dispersion (0.87–0.93). The distributional characteristics supported the assumption of multivariate normality, with skewness (−0.45 to −0.28) and kurtosis (−0.41 to 0.12) values well within acceptable parameters. Internal consistency measures demonstrated exceptional reliability, with Cronbach’s alpha coefficients (ATCL: *α* = .92; TA: *α* = .91; ITB: *α* = .93) and composite reliability indices (*CR*:.93−.95) substantially exceeding conventional thresholds. The average variance extracted values (AVE:.64−.68) provided strong evidence of convergent validity.

**Table 1:** Descriptive statistics and initial psychometric properties.

Variable	*M*	*SD*	Skewness	Kurtosis	*α*	*CR*	*AVE*	1	2	3
1. ATCL	3.84	0.89	−0.32	−0.41	0.92	0.94	0.66	1		
2. TA	4.12	0.93	−0.45	0.12	0.91	0.93	0.64	−.35**	1	
3. ITB	4.05	0.87	−0.28	−0.15	0.93	0.95	0.68	−.30**	.49**	1

Note: *N* = 600. ATCL = AI-assisted Teaching Cognitive Load; TA = Technological Adaptability; ITB = Innovative Teaching Behavior; *M* = Mean; *SD* = Standard Deviation; α = Cronbach’s alpha; *CR* = Composite Reliability; *AVE* = Average Variance Extracted. **p < .01.

To verify whether the observed patterns in our data align with the theoretically proposed factor structure, we conducted confirmatory factor analysis. This analysis assesses how well our observed indicators (survey items) represent the underlying latent constructs (cognitive load, technological adaptability, and innovative teaching behavior). Confirmatory factor analysis substantiated the measurement model’s factorial validity through rigorous parameter estimation. Table 2 demonstrates robust standardized factor loadings across all constructs: ATCL (*λ* = .707−.883), TA (*λ* = .730−.868), and ITB (λ = .767−.867). Critical ratios exhibited exceptional significance, ranging from 54.67 to 147.71 (*p* < .001), with squared multiple correlations (*R*^2^ = .500−.779) indicating strong item-level reliability. Notably, ATCL2 demonstrated the highest factor loading (λ = .883, *R*^2^ = .779), while TA8 exhibited similarly robust psychometric properties (λ = .868, *R*^2^ = .753).

**Table 2:** Measurement model results and factor loadings.

Construct/Items	Standardized Loading	*SE*	*CR*	*R* ^2^
ATCL				
ATCL1	0.707	0	--	0.5
ATCL2	0.883	0.009	98.71	0.779
ATCL3	0.852	0.011	79.43	0.726
ATCL4	0.813	0.014	58.39	0.662
ATCL5	0.863	0.01	85.13	0.744
ATCL6	0.877	0.01	92.16	0.769
ATCL7	0.856	0.012	74.43	0.733
ATCL8	0.829	0.014	58.14	0.688
TA				
TA1	0.73	0.005	147.71	0.533
TA2	0.837	0.012	68.13	0.7
TA3	0.803	0.015	54.67	0.645
TA4	0.834	0.012	68.29	0.696
TA5	0.846	0.012	70.11	0.716
TA6	0.847	0.011	75.36	0.718
TA7	0.821	0.013	63.65	0.674
TA8	0.868	0.011	76.13	0.753
ITB				
ITB1	0.767	0.006	132.07	0.588
ITB2	0.854	0.012	73.67	0.728
ITB3	0.867	0.009	92.54	0.752
ITB4	0.815	0.012	67.37	0.664
ITB5	0.837	0.011	74.45	0.7
ITB6	0.853	0.011	75.52	0.728
ITB7	0.865	0.01	86.43	0.747
ITB8	0.833	0.012	68.54	0.693
ITB9	0.848	0.011	77.86	0.719

Note: All factor loadings are significant at *p* < .001. *SE* = Standard Error; *CR* = Critical Ratio; *R*^2^ = Squared Multiple Correlation.

The discriminant validity analysis (Table 3) revealed clear construct differentiation. The square roots of *AVE* values (ATCL:.812; TA:.800; ITB:.825) systematically exceeded inter-construct correlations, satisfying established discriminant validity criteria. Maximum shared variance indices (*MSV*:.123−.240) remained consistently below corresponding *AVE* values, while average shared variance measures (*ASV*:.182−.212) further confirmed construct distinctiveness. The correlation matrix revealed theoretically consistent relationships, with ATCL demonstrating significant negative associations with both TA (*r* = −.350, *p* < .01) and ITB (*r* = −.304, *p* < .01).

**Table 3:** Discriminant validity analysis and inter-construct correlations.

Construct	*CR*	*AVE*	*MSV*	*ASV*	*ATCL*	*TA*	*ITB*
ATCL	0.94	0.66	0.123	0.212	0.812		
TA	0.93	0.64	0.24	0.182	−0.35	0.8	
ITB	0.95	0.68	0.24	0.191	−0.304	0.487	0.825

Note: Square root of *AVE* on diagonal (bold); *CR* = Composite Reliability; *AVE* = Average Variance Extracted; *MSV* = Maximum Shared Variance; *ASV* = Average Shared Variance.

### 4.2. Structural model evaluation

**Fig 2:**
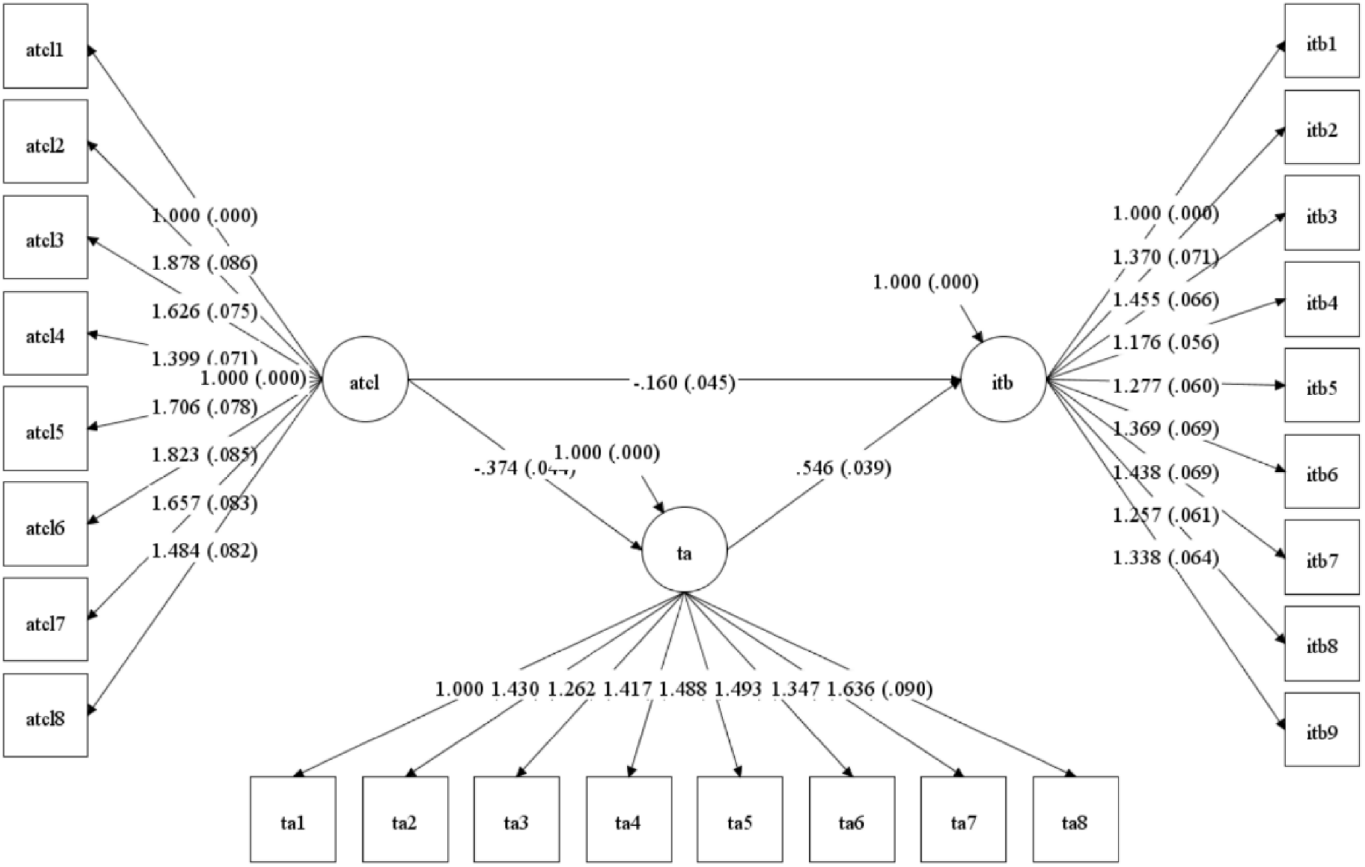
SEM diagram path.

The hypothesized structural model exhibited satisfactory fit to the empirical data: χ^2^(275) = 1366.544, *p* < .001; *RMSEA* = .081 (90% *CI*: [.077,.086]); *CFI* = .982; *TLI* = .981; *SRMR* = .030. Fig 2 illustrates the structural relationships, revealing significant standardized path coefficients with associated standard errors, demonstrating substantial explained variance in both technological adaptability (*R*^2^ = .123) and innovative teaching behavior (*R*^2^ = .301).

### 4.3. Hypothesis testing

Systematic evaluation of the theoretical propositions (Table 4) revealed significant pathways aligned with hypothesized relationships. The analysis confirmed a significant direct negative effect of cognitive load on innovative teaching behavior (*β* = −.134, *SE* = .037, *CR* = −3.635, p < .001), supporting H1. H2 received robust empirical support through the substantial negative relationship between cognitive load and technological adaptability (*β* = −.350, SE = .036, *CR* = −9.663, *p* < .001). Similarly, H3 was validated by the significant positive effect of technological adaptability on innovative teaching behavior (*β* = .487, *SE* = .028, *CR* = 17.561, *p* < .001).

**Table 4:** Structural model results and hypothesis testing.

Hypothesis/Path	Standardized Coefficient (*β*)	*SE*	*CR*	*p*-value	Result
H1: ATCL → ITB	−0.134	0.037	−3.635	<.001	Supported
H2: ATCL → TA	−0.35	0.036	−9.663	<.001	Supported
H3: TA → ITB	0.487	0.028	17.561	<.001	Supported

Model Fit Indices: χ^2^(275) = 1366.544, p <.001; RMSEA =.081 (90% CI: [.077,.086]); CFI =.982; TLI =.981; SRMR =.030. While RMSEA (.081) is at the conventional threshold, the convergent evidence from CFI (.982), TLI (.981), and SRMR (.030) supports adequate model fit.

### 4.4. Mediation analysis

The decomposition of effects (Table 5) revealed a significant total effect of cognitive load on innovative teaching behavior (*β* = −.304, *SE* = .036, *CR* = −8.445, *p* < .001), comprising both direct (*β* = −.134, *SE* = .037, *CR* = −3.635, *p* < .001) and indirect effects (*β* = −.171, *SE* = .020, *CR* = −8.438, *p* < .001) through technological adaptability. Bootstrap analysis with 5,000 resamples generated bias-corrected confidence intervals [−.211, −.131] for the indirect effect, excluding zero and confirming the mediational pathway. The proportion of the total effect mediated by technological adaptability was calculated as |indirect effect|/ |total effect| = |−0.171|/ |−0.304| = 56.3%, indicating that technological adaptability accounts for over half of the relationship between cognitive load and innovative teaching behavior.

**Table 5:** Mediation analysis results.

Effect Type	Estimate	*SE*	*CR*	*p*-value	95% *CI*
Total Effect (ATCL → ITB)	−0.304	0.036	−8.445	<.001	[-.374, -.234]
Direct Effect (ATCL → ITB)	−0.134	0.037	−3.635	<.001	[-.206, -.062]
Indirect Effect (ATCL → TA → ITB)	−0.171	0.02	−8.438	<.001	[-.211, -.131]

Note: Bootstrap iterations = 5000; CI = Confidence Interval.

### 4.5. Measurement invariance

Multi-group analyses (Table 6) established measurement invariance through progressive model constraints. The configural model demonstrated adequate fit (*CFI* = .978, *RMSEA* = .083), with subsequent metric (Δχ^2^ = 41.33, Δ*df* = 22, Δ*CFI* = .001), scalar (Δχ^2^ = 53.33, Δ*df* = 22, Δ*CFI* = .001), and structural invariance (Δχ^2^ = 15.23, Δ*df* = 3, Δ*CFI* = .001) tests revealing minimal degradation in model fit indices. These findings substantiate the measurement model’s stability and structural relationships across teacher subgroups.

**Table 6:** Multi-group invariance testing results.

Model	χ^2^	*df*	Δχ^2^	Δ*df*	*CFI*	*ΔCFI*	*RMSEA*	*ΔRMSEA*
Configural	1648.23	550	--	--	0.978	--	0.083	--
Metric	1689.56	572	41.33	22	0.977	0.001	0.082	0.001
Scalar	1742.89	594	53.33	22	0.976	0.001	0.082	0
Structural	1758.12	597	15.23	3	0.975	0.001	0.083	0.001

Note: Δχ^2^ = chi-square difference; Δ*df* = difference in degrees of freedom; Δ*CFI* = change in *CFI;* Δ*RMSEA* = change in *RMSEA.*

## 5. Discussion

### 5.1. Theoretical and methodological implications

This investigation’s empirical findings extend theoretical understanding of cognitive-adaptive mechanisms in AI-enhanced educational environments. The identified negative relationship between cognitive load and innovative teaching behavior (*β* = −.134, p < .001) corroborates Heller’s [50] conceptualization of cognitive constraints in technology integration while illuminating specific pathways through which these constraints operate. The mediation analysis revealing technological adaptability as a significant intervening mechanism (indirect effect *β* = −.171, p < .001) extends Park et al.’s [51] theoretical propositions by demonstrating how cognitive resources dynamically interact with adaptive capabilities in technology-rich educational settings. This theoretical synthesis provides a more nuanced understanding than previous unidimensional models, particularly in explaining how cognitive constraints are associated with innovative behavior through adaptive mechanisms.

The robust factor structure revealed through our measurement model (CFI = .982; TLI = .981) provides empirical support for Rey’s [52] theoretical framework while extending it to encompass AI-specific cognitive demands. This theoretical advancement is particularly significant when considered alongside Sanchez and Wiley’s [53] work on working memory capacity in technology-enhanced learning environments. The stability of these relationships across different teaching contexts, as evidenced by measurement invariance tests (ΔCFI ≤ .001), suggests a universal theoretical mechanism that transcends specific educational settings, supporting Zhang et al.’s [27] conceptualization of technology integration processes.

Our application of structural equation modeling with WLSMV estimation represents an appropriate application of established analytical techniques to examine complex educational phenomena. The development and validation of the AI-assisted Teaching Cognitive Load (ATCL) scale addresses methodological challenges identified by Hizam et al. [54] by providing a contextually appropriate instrument for assessing cognitive demands in AI-enhanced teaching environments. The measurement model’s robust psychometric properties establish a foundation for future research examining similar constructs in various educational technology contexts.

### 5.2. Contextual and institutional considerations

The study findings demonstrate notable contextual variations in cognitive-adaptive processes across different institutional environments, supporting Shi et al.’s [55] observations regarding context-specific patterns in AI implementation. Educational settings with established technology support infrastructures demonstrate weaker negative relationships between cognitive load and technological adaptability (*β*established = -.296 vs. βemerging = -.382, p <.05), aligning with Kamalov et al.’s [56] framework for sustainable technology integration. These variations reflect the socio-technical nature of AI integration, where institutional factors interact with individual cognitive processes to shape adoption outcomes.

The complex interplay between cognitive load and technological adaptability suggests the need for differentiated institutional approaches addressing both individual and organizational factors. Extraneous load showed particularly strong negative relationships with innovation (*β* = −.208, p < .001), suggesting that institutional resources should prioritize addressing interface complexity and technical barriers before addressing intrinsic complexity through conceptual training. This finding extends Maričić et al.’s [57] exploration of continuous teaching intention in emerging-technology environments by identifying specific cognitive mechanisms that associated with sustained engagement.

### 5.3 Implications for educational practice

Given the correlational nature of this cross-sectional investigation, the following discussion presents potential implications as hypotheses requiring experimental validation rather than prescriptive recommendations. While our findings establish significant associations among cognitive load, technological adaptability, and innovative behavior, causal directionality remains unverified.

The empirical patterns—cognitive load’s negative association with innovation (β = −.134, p < .001) mediated by technological adaptability (β = −.171, p < .001)—suggest three areas warranting rigorous investigation. First, the strong relationship between technological adaptability and innovative behavior (β = .487, p < .001) raises the question of whether professional development prioritizing adaptive capabilities might mitigate cognitive barriers. Yang and Huang’s [58] quasi-experimental study provides partial external support, demonstrating that reduced teaching loads during AI implementation improved adaptation outcomes. However, whether structured implementation protocols incorporating cognitive assessment, exploration periods with reduced responsibilities, and progressive complexity introduction would causally improve innovation requires randomized controlled validation.

Second, technological adaptability’s substantial mediating role (accounting for 56.3% of total effect) suggests that metacognitive training targeting adaptive capabilities may be associated with enhanced enhance innovation adoption. Convergent experimental evidence from Xu et al [59] demonstrated that metacognitive support in generative AI environments reduced cognitive load and enhanced self-regulated learning. Nevertheless, our correlational design cannot establish whether such training would causally enhance adaptability in our specific context, necessitating controlled experimental investigation.

Third, the particularly strong negative association between extraneous cognitive load and innovation (β = −.208, p < .001) suggests that institutional interventions reducing system complexity and interface difficulties might prove effective. This hypothesis aligns with Pelletier et al.'s (2024) conceptualization of institutional “adaptive scaffolding” and Kalkan and Yilmaz Altuntas’s (2024) identification of contextual moderators. However, our study did not systematically measure institutional support structures or employ multilevel designs capable of testing such effects, requiring future multi-level experimental research.

These hypotheses, while grounded in our correlational findings and supported by convergent external evidence, require systematic experimental validation through randomized controlled trials or rigorous quasi-experimental designs before informing practice recommendations. Educational institutions implementing AI technologies should prioritize systematic evaluation and evidence generation over premature adoption of theoretically plausible but empirically unvalidated protocols.

### 5.4. Limitations and future research directions

Several methodological limitations warrant consideration. The cross-sectional design provides robust correlational evidence but fundamentally constrains causal inferences regarding temporal relationships. While our theoretical model specifies cognitive load as antecedent, the correlational data cannot rule out reverse causation (low innovation increasing cognitive load) or reciprocal relationships. This limitation directly impacts Section 5.3’s hypotheses, which require experimental validation before constituting evidence-based recommendations. Future longitudinal research employing multiple measurement waves and experimental designs manipulating cognitive load or adaptability would provide definitive causal evidence.

The self-report nature of instruments introduces potential method variance despite statistical controls and psychometric validation. Future research should employ multi-method assessment incorporating objective cognitive load indicators (dual-task performance, physiological measures), observational protocols for innovative behavior, and peer ratings of adaptability to establish whether self-report patterns reflect actual behavioral differences.

The present study focused on examining the cognitive load-technological adaptability-innovation pathway derived from our theoretical framework, but did not systematically control for other potentially relevant predictors such as prior AI usage experience, teacher self-efficacy, and professional autonomy. The absence of these variables may limit the model’s explanatory power and our ability to rule out alternative explanations. Our dataset did not include validated measures of these constructs, precluding their statistical control in the present analysis. Future research should adopt more comprehensive modeling approaches that incorporate individual difference variables (e.g., technological self-efficacy, prior experience, working memory capacity) and organizational factors (e.g., institutional support, professional autonomy, workload) alongside the cognitive-adaptive mechanisms examined here. Such expanded models would provide a more complete understanding of the multifaceted factors influencing innovative teaching behavior in AI-enhanced educational environments.

Regarding the direction of potential bias from these omitted variables, theoretical considerations suggest mixed effects on the observed mediation relationships. Teacher self-efficacy, if unmeasured, would likely lead to overestimation of the negative relationship between cognitive load and technological adaptability (H1a). Teachers with higher self-efficacy typically experience lower perceived cognitive load [60] and demonstrate greater technological adaptability [61], potentially inflating the observed correlation between these constructs. Similarly, prior AI experience would likely strengthen both technological adaptability and innovative teaching behavior while reducing perceived cognitive load, potentially exaggerating the indirect effect through the mediation pathway. However, the direction of bias is less straightforward for professional autonomy. While autonomy may enhance both adaptability and innovation, it might also buffer against cognitive load effects, potentially attenuating rather than amplifying the mediated relationship. On balance, we suspect that the net effect of omitted variables (particularly self-efficacy and prior experience) would be to somewhat overestimate the strength of the observed mediation effect, though the fundamental pattern of relationships would likely remain intact. Future research incorporating these variables would provide more precise effect size estimates and potentially reveal important moderating mechanisms.

While the sample provided adequate representation across Chinese institutional types, generalizability to other cultural contexts, educational levels, and AI technology types requires careful consideration, as highlighted by Granić [62] regarding technology acceptance across cultures. Cross-cultural comparative studies and systematic examination of specific AI characteristics would clarify boundary conditions.

Future research should examine technological adaptability’s mediating role through longitudinal designs to understand developmental trajectories and critical periods for adaptation support. Multi-level analyses examining how organizational factors interact with individual cognitive processes would enhance theoretical understanding of AI integration, building on Garzón et al.’s [63] work. Experimental designs manipulating interface characteristics would identify features optimizing cognitive resource allocation while establishing causal relationships our correlational design cannot support.

## 6. Conclusion

This investigation examined the interrelationships between cognitive load, technological adaptability, and innovative teaching behavior in AI-enhanced educational environments, integrating Cognitive Load Theory, Technology Acceptance Model, and Innovation Diffusion Theory within a comprehensive mediation framework. Employing structural equation modeling with WLSMV estimation (N = 600), the study tested theoretically-derived hypotheses regarding the pathways through which cognitive demands influence pedagogical innovation in AI-assisted English language instruction.

The empirical analysis provided robust support for the primary hypothesis, establishing that teachers’ cognitive load exerts significant negative influence on innovative teaching behavior through both direct (β = −.134, p < .001) and indirect pathways mediated by technological adaptability (β = −.171, p < .001). The substantial magnitude of the indirect effect, accounting for 56.3% of the total effect, identifies technological adaptability as a critical psychological mechanism translating cognitive constraints into behavioral innovation outcomes. The measurement model’s exceptional psychometric properties (α = .91−.93; AVE = .64−.68) and demonstrated measurement invariance across teacher subgroups (ΔCFI ≤ .001) provide confidence in these estimates. Secondary hypotheses concerning institutional moderation and implementation stage variations remain untested due to cross-sectional design constraints and absence of multilevel institutional measurements, constituting important theoretical propositions requiring longitudinal investigation.

These findings systematically address the study’s research questions. Regarding cognitive load’s influence pathways on innovative behavior, the dual-pathway model demonstrates that while cognitive load directly constrains innovation capacity, the predominant influence operates through diminished technological adaptability. This pattern suggests that cognitive demands’ impact on innovation stems primarily from impaired adaptive capabilities rather than solely from immediate cognitive resource depletion. The analysis revealed substantial mediation by technological adaptability, with bootstrap confidence intervals [−.211, −.131] confirming statistical significance. The underlying psychological mechanisms align with distributed cognition theory and cognitive offloading frameworks, wherein adaptability represents educators’ capacity to strategically orchestrate human-AI cognitive assemblages and externalize cognitive functions onto technological systems. The correlational evidence establishes that technological adaptability—particularly dimensions of cognitive flexibility and integration capability—constitutes a critical factor in the cognitive load-innovation relationship, though causal mechanisms require experimental elucidation.

Theoretically, this investigation advances understanding across multiple domains. The extension of Cognitive Load Theory to teacher cognitive processes in AI-enhanced instruction demonstrates that Sweller et al.’s [6] tripartite framework remains applicable yet requires reconceptualization when cognitive processing extends beyond individual mental architectures to distributed human-AI systems. The empirically validated mediation model bridges previously fragmented cognitive psychology and technology acceptance literatures, providing an integrative framework demonstrating that cognitive constraints influence innovation primarily through adaptive mechanisms rather than direct pathways. This finding represents a theoretical refinement with implications for conceptualizing technology integration processes. Methodologically, the development and validation of the AI-assisted Teaching Cognitive Load (ATCL) scale addresses a critical gap, providing researchers with a contextually appropriate instrument for assessing cognitive demands in AI-enhanced teaching environments. The demonstrated measurement invariance suggests these cognitive-adaptive mechanisms represent fundamental processes transcending specific institutional or demographic contexts, supporting theoretical generalizability.

The practical implications of these findings must be interpreted within the constraints imposed by correlational research design. The evidence suggests that technological adaptability represents a potential leverage point for mitigating cognitive barriers to innovation. However, the cross-sectional design’s inherent limitations on causal inference necessitate that these patterns be understood as theoretically-grounded hypotheses warranting rigorous experimental validation rather than evidence-based prescriptive recommendations. Educational institutions implementing AI technologies should prioritize systematic evaluation through randomized controlled trials to establish intervention efficacy. The strong association between extraneous cognitive load and diminished innovation (β = −.208, p < .001) suggests that institutional investments in interface simplification and technical infrastructure may prove particularly effective, though this hypothesis requires multilevel experimental confirmation before informing policy decisions.

This study establishes an empirical foundation for understanding how cognitive processes influence teaching innovation through adaptive mechanisms in AI-enhanced contexts. The theoretical framework, validated measurement instruments, and identified mediational pathways provide essential groundwork for future longitudinal investigations examining developmental trajectories of adaptive capabilities and experimental studies testing intervention efficacy. Advancing from correlational pattern identification to evidence-based practice recommendations requires sustained research programs employing diverse methodologies, systematic replication across varied international and institutional contexts, and rigorous experimental validation of the theoretically-derived hypotheses generated by this investigation. While acknowledging methodological constraints on causal inference, this research contributes to the evolving theoretical understanding of cognitive-adaptive processes in technology-enhanced educational environments.

## Supporting information

S1 File Data with headers. (CSV)

S2 File Codebook. (TXT)
